# Responses to active stand test predict 12-year incident cardiovascular disease and mortality

**DOI:** 10.1038/s43856-025-01253-3

**Published:** 2025-12-13

**Authors:** Belinda Hernández, Adam H. Dyer, Cathal McCrory, Louise Newman, Mark Ward, Ciaran Finucane, Rose Anne Kenny

**Affiliations:** 1 The Irish Longitudinal Study on Ageing, Trinity College Dublin, Dublin, 2 Ireland; 2 Discipline of Medical Gerontology, School of Medicine, Trinity College, Dublin, Ireland; 3 National Center for Pharmacoeconomics, St James Hospital, Dublin, Ireland; 4 Department of Age-Related Healthcare, Tallaght University Hospital, Dublin, Ireland; 5 Mercer’s Institute for Successful Ageing, St. James’s Hospital, Trinity College, The University of Dublin, Dublin, Ireland; 6 Deptartment of Medical Physics and Bioengineering, St. James’s Hospital, Dublin, Ireland

**Keywords:** Cardiology, Biomarkers

## Abstract

**Background:**

An integrated haemodynamic response during standing may serve as a marker of neuro-cardiovascular function. Individual components of both heart rate (HR) and blood pressure (BP) responses to active stand (AS) have been linked with cardiovascular disease (CVD) and mortality. We hypothesised that integrating BP/HR information from the entire haemodynamic response curve may uncover otherwise unknown associations with both CVD and mortality.

**Methods:**

Beat-to-beat measurements of dynamic HR/BP responses to AS were conducted in 4336 individuals (61.5 ± 8.2 years; 53.7% female). We assessed longitudinal associations between entire HR/BP response curves during AS, incident CVD and mortality over 12 years, and cross-sectional association with CVD on an independent clinical cohort. Functional Principal Components Analysis was applied to response curves, and their association with CVD and mortality was assessed.

**Results:**

In multivariable models, components with higher systolic BP (SBP) before AS and blunted recovery of SBP during AS are associated with all-cause mortality over 12-years (Hazard Ratio [HR]: 1.14; 1.04, 1.26; p = 0.007). Components with higher baseline/peak HR and lower HR from 30 seconds post stand are associated with lower Hazard of cardiovascular deaths (HR: 0.78; 0.64, 0.95; p = 0.013). Impaired recovery of DBP from 35 seconds onward is associated with CVD in a validation cohort (Odds Ratio: 0.65; 0.17, 0.88).

**Conclusions:**

We observe distinct relationships between HR/BP responses to AS and 12-year incident CVD and mortality. BP recovery and CVD are also associated in an independent clinical validation cohort. Integrating the entire haemodynamic response may reveal more nuanced relationships between HR/BP responses to AS, CVD and mortality - serving as an integrative marker of neuro-cardiovascular health in midlife and beyond.

## Plain language summary

Standing up provides an effective test of the body’s nerve and heart regulation. This study investigated whether a person’s heart rate (HR) and blood pressure (BP) response to standing could better predict future risk of death and heart disease than standard single-measure summaries. Cardiovascular measures of ~4300 adults during a standing test were analysed to study the link between their response to standing, cardiovascular disease and mortality. Key findings revealed that high BP before standing and slow recovery of BP after standing were strongly linked to a higher risk of death. Additionally, poor BP and HR recovery were associated with future heart disease. These results suggest that using all available data in response to standing offers a more sensitive marker of cardiovascular health than existing measures.

## Abstract (web summary)

Hernández et al. examine the link between heart rate and blood pressure response to active standing and future cardiovascular disease (CVD). Findings reveal distinct haemodynamic patterns linked to higher mortality and CVD risk, highlighting its potential as a marker for cardiovascular health.

## Introduction

Adults typically stand from sitting or lying supine between 50-60 times per day^1,2^. The haemodynamic response to standing (or orthostasis) is a mild physiological stressor requiring integrated neuro-cardiovascular function, which can be readily assessed clinically through measurement of blood pressure (BP) and heart rate (HR) responses to formal active stand (AS) in the clinic^3,4^. In addition to its role in assessing for orthostatic hypotension (OH) and autonomic dysfunction in older adults, the haemodynamic response to active stand can give an excellent indicator of the integrity of short-term cardiovascular and neurogenic responses^5–7^.

OH is defined as a drop of ≥20 mmHg systolic/ ≥ 10 mmHg diastolic BP on standing from supine. In addition to being a significant cause of falls and syncope in older adults^8–10^, OH has been associated with cardiovascular/cerebrovascular disease^11–17^, cognitive impairment/dementia^18–21^, depression^22^, gait impairment^23^ and frailty^24,25^. These associations highlight the role of haemodynamic response to active stand as an important indicator of intact homoeostatic neuro-cardiovascular function and as a potentially important marker of physiological health in older adults.

Importantly, OH and impaired BP responses to AS have also been associated with an increased risk of mortality in community-dwelling older adults^11,26–32^, although negative studies also exist^33,34^. Nearly-all studies have used sphygmomanometer measurement of BP at discrete timepoints, such as 40 or 60 seconds post-stand. Such measurements fail to account for the broader range of haemodynamic responses to active stand. Broader responses can be readily assessed using non-invasive beat-to-beat finometry, which gives waveform measurements similar to those from invasive intra-arterial monitoring, capturing dynamic changes often missed with other techniques^35–38^. Using beat-to-beat finometry also enables classification of different BP phenotypes in response to AS which may differ in their associations with important clinical outcomes such as mortality^38,39^.

In addition to BP response to AS, dynamic changes in HR response to AS may also be associated with mortality. Research has clearly demonstrated an association between HR Recovery (HRR) following exercise and increased mortality^40^. However, studies vary in stressor applied (most frequently treadmill exercise) and typically assess recovery at 1–5 minute period post-exercise cessation^41,42^. Given that standing is a potent and ubiquitous stressor which can be performed by anyone who is functionally mobile, comparatively few studies have examined the association between HR responses to AS and subsequent mortality in community-dwelling older adults^43,44^.

HR rapidly increases in the immediate period post-stand, peaking at around 10 seconds in order to counteract gravitational forces on BP. This is felt to result from an abrupt inhibition of vagal activity, after which HR rapidly declines reflecting parasympathetic reactivation^45,46^. HR peak and decline typically takes place in the immediate 30-second period post-stand. Previous work has demonstrated that a slower speed of HRR may be a strong predictor of mortality in older adults^47^. Similarly, studies have demonstrated significant associations between higher baseline HR before AS and in the 30–60 second post-stand time period and mortality^48^. Whilst the association between an increased resting HR and both overall and cardiovascular mortality has been firmly established in the literature^49–51^, the relationship of HRR to AS and mortality has been less well explored.

Despite associations between both BP and HR responses to AS and increased mortality in older adults, to the best of our knowledge no study to date has integrated these responses in the same study. Whilst most studies consider BP/HR responses at a single timepoint/scalar summaries of responses at short intervals, none have considered the complete haemodynamic response curve to AS. In the current study embedded within The Irish Longitudinal Study of Ageing (TILDA), we used functional Principal Components Analysis (FPCA) to examine the determinants of variability in continuous beat-to-beat haemodynamic response to AS and their association with both 12-year incident CVD and 12-year mortality. We also show the flexibility and utility of our FPCA model to predict CVD on a separate clinical cohort validation study of patients from the Falls and Syncope Unit from St James’ Hospital, Dublin.

Overall, we find that higher systolic blood pressure before standing and blunted recovery of SBP during standing is associated with all-cause mortality over 12-years. Higher baseline and peak HR combined with lower HR from 30 seconds post stand are associated with lower mortality due to circulatory causes. Furthermore, components related to lower heart rate and elevated post stand stroke volume index are associated with 12-year incident CVD. These findings persist on robust covariate adjustment. To showcase the clinical utility of these models we validate our model on a separate clinical cohort of *n* = 347 patients with existing CVD and find that components for impaired recovery of diastolic blood pressure are associated with existing CVD. We also show that consensus measures such as OH 60 seconds after stand and baseline blood pressure on their own are not associated with 12-year mortality. However, principal components associated with higher baseline systolic blood pressure and impaired blood pressure recovery are. This shows that integrating information from the entire haemodynamic response curve may reveal more subtle relationships between these responses, CVD and mortality, serving as an integrative marker of neuro-cardiovascular health in middle and older aged adults.

## Methods

### Study approval, setting and participants

#### The Irish Longitudinal Study on ageing

Data were obtained from Wave 1 (2010) of The Irish Longitudinal Study on Ageing (TILDA), a prospective nationally-representative study of community-dwelling older adults in Ireland. Participants with dementia and/or cognitive impairment were excluded at baseline. All data from the active stand test were obtained in a health centre with assessments administered by trained nurses^52,53^. Informed consent was provided by the participant’s for the TILDA study and was approved by the Faculty of Health Sciences Research Ethics Committee at Trinity College Dublin and adhered to the Declaration of Helsinki. A total of 4336 participants were included in the analysis from an initial sample of 4956. A flow chart including inclusion criteria are shown in Supplementary Fig. 1.

### The Active Stand

The Active Stand (AS) test in TILDA was performed by participants who completed the wave 1 TILDA health centre assessment. Briefly, participants lay supine for ~10 minutes before being asked to stand. They then remained standing for two minutes. The test was supervised by a research nurse who assisted with standing as necessary. Continuous beat-to-beat measurements of HR, systolic blood pressure (SBP), diastolic blood pressure (DBP) and stroke volume were captured at 1 Hz from 30 seconds prior to standing to 2 minutes post standing (resulting in 151 measurements per person) using a Finometer MIDI device (Finapres Medical Systems BV, Amsterdam, the Netherlands). A finger cuff was placed on the middle or proximal phalanx of a finger (as per manufacturer instructions) on the left hand. Calibration was performed using Physiocal™, while hydrostatic pressure differences were corrected for using the system’s height correction unit.

Absolute HR was analysed as this is the only signal which can be accurately captured by the Finometer in absolute terms^54^, for all other signals, the change from baseline (60 to 30 seconds before standing) was analysed. SV was normalised by body surface area prior to analysis. Therefore, this signal describes the change from baseline SV per square metre of body surface area and is referred to as the stroke volume index (SVI) henceforth.

### Mortality status

**Fig. 1:**
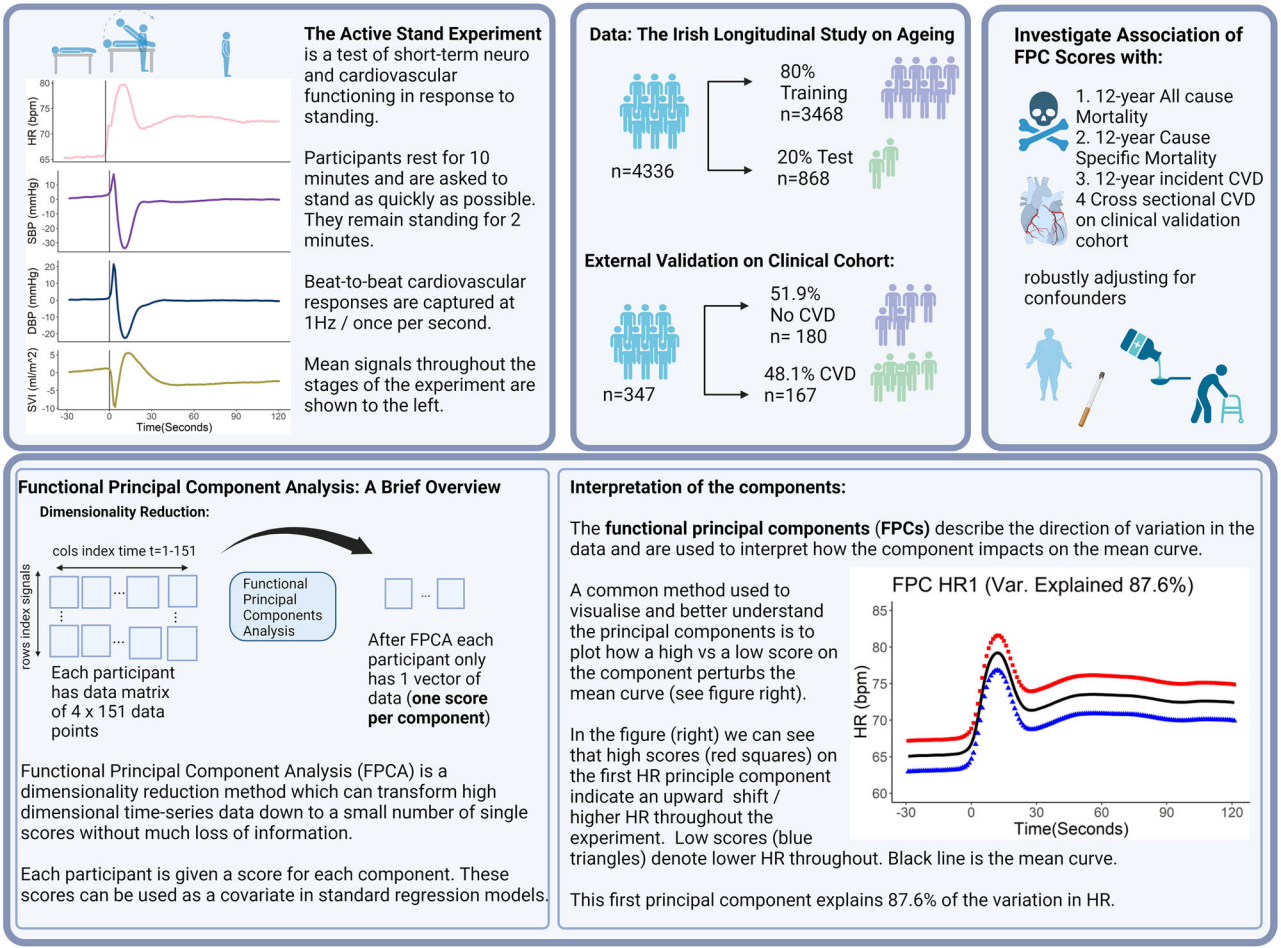
Methodology overview. Overview of the methodology used to investigate the association between haemodynamic responses to standing and 12-year mortality and incident cardiovascular disease.

In Ireland, all deaths are registered through the General Register Office (GRO). Mortality status and cause of death were determined by inspection of death certificates over a ~12-year follow-up using data linkage between TILDA and the GRO. The methodology and procedure for determining cause of death has been documented elsewhere^55^. Data are available for all reported deaths of TILDA participants up to 31st January 2022. Deaths unofficially reported to TILDA by family members but not yet included in the GRO were excluded as the exact date and cause of death was not ascertainable as were deaths by suicide or deaths due to accidents (*n* = 29 see Fig. 1).

### Clinical validation cohort for cardiovascular disease associations

A cross-sectional clinical validation study of patients referred to the Falls and Syncope Unit (FASU) in St James’ Hospital Ireland was used to assess the association of the functional principal component model for CVD on an independent cohort. As the study was cross-sectional, mortality data was not available in the replication cohort. Briefly, patients aged 50 and over who had been referred to the FASU due to reporting issues with episodes of dizziness, falls or syncope were recruited into the study. Patients with a diagnosis of cognitive impairment or who were unable to provide informed consent or patients who were unable to perform the active stand experiment were excluded from participation. The study was approved from the Tallaght University Hospital/St. James’s Hospital Joint Research Ethics Committee (REC:2018-08,CA,16).

### Statistics and reproducibility

#### Univariate analysis

For descriptive summaries, categorical variables were expressed as percentages and compared between groups using a Chi-Squared test. Continuous variables were expressed as a mean and standard deviation and compared between groups using a one-way ANOVA.

For univariate summaries of the peripheral haemodynamic signals, group means were compared using functional ANOVA. For more details on this methodology see Supplementary Methods 1.

#### Functional principal components analysis

FPCA is an extension of the well-known dimensionality reduction technique principal components analysis (PCA) for functional data (in this case haemodynamic responses as a function of time). In the following analyses, functional principal components analysis (FPCA) was performed separately on each of the peripheral haemodynamic signals. The number of principal components which explained 90% of the variability in the data were retained. A total of 2 functional principal components (FPCs) were retained for HR, 5 for SBP, 6 for DBP and 4 for SVI. FPC scores associated with these components were then extracted and used as covariates in the models for CVD and mortality described in the following section. A brief explanation of FPCA and study design can be seen in Fig. 1 see also Supplementary Methods 2 for a more detailed methodological description and^56–58^ for further examples.

#### Multivariable survival models

A Cox PH model was implemented to model all-cause mortality with age of death as the outcome variable. Those who survived were right-censored to their age as of 31st Jan 2022. The survival status of all participants is known as of 31st January 2022, regardless of attrition from the TILDA study due to data linkage with the GRO. Because of the high correlation between the response of SBP and DBP to standing, only the results for SBP are shown. However, including DBP in the models does not change the conclusions of this work. The proportionality assumption of the Cox Proportional Hazard model was verified by assessing the scaled Schoenfeld residuals over time. For cause-specific mortality, a competing risk analysis using the Fine and Grey method was used. Final models were selected using backward variable selection.

In all cases, models were trained on a random 80% of the data and tested on the remaining 20% which was independent of the model training. To assess the model performance of both the all-cause and cause-specific models on independent data; the time-dependent AUC and 95% confidence interval were calculated using the 20% test dataset.

#### Multivariable models for CVD

Incident CVD in the TILDA study was defined as presence of at least one of the following conditions: congestive heart failure, angina, heart attack or atrial fibrillation at follow up waves 2–6 ( ~ 12 year follow up). Participants with CVD at baseline were removed from the analysis. In total, 2419 participants who didn’t have CVD at wave 1 were included in the analysis of incident CVD. To model CVD incidence, an elastic net logistic regression was trained on 80% of the data and all performance metrics were assessed using 20% test data. For more information see Supplementary Methods 3. Final models were selected using backward variable selection.

In the FASU clinical validation cohort, CVD was defined as the presence of any of the following conditions: transient ischaemic attack, stroke, carotid stenosis, ischaemic heart disease, angina, myocardial infarction, atrial fibrillation, heart failure, diabetes. To ensure uniformity of methodologies across datasets the same methodology as described for incident CVD above was also used on this cohort. Age, sex, BMI, smoking history, hypertension and high cholesterol were controlled for.

All statistical analysis was performed using the R version 4.2.3.

## Covariates

The following covariates known to be associated with mortality and incident CVD were controlled for in all models based on TILDA data: age; sex (binary); education (Primary/Secondary/Third level); smoking history (Never, Former, Current); BMI (numeric); hypertension (binary); high cholesterol (binary), depression, anxiety or psychosis (binary) and frailty.

Hypertension was defined as systolic blood pressure >140 mmHg and/or diastolic blood pressure >90 mmHg and/or use of any of the following medications: ATC codes C02, C03, C07, C08, C09. Presence of high cholesterol was defined as Total Cholesterol ≥5 mmol/L and/or use of ATC C10 (excluding C10AX06). Presence of depression, anxiety or psychosis was defined as use of any of the following medications: N05BA, N05AE, N05CD, N05CF, N06A, N05A) and/or a score ≥11 on the Hospital Anxiety and Depression Scale (HADS-A) and / or a score of ≥16 on the Centre for Epidemiological Studies Depression Scale (CES-D). Frailty was defined using the 32-item frailty index^59^ which captures information regarding disability, symptoms, signs and diseases affecting a range of organ systems. The FI has previously been operationalised in TILDA and characterises participants into robust (FI score <0.1), pre-frail (0.1 ≤ FI score <0.25) or frail (FI score ≥ 0.25) based on their index scores^60,61^.

For all-cause mortality further adjustment for presence of CVD and neurodegenerative disease at baseline were accounted for. Specifically, a history of angina, heart attacks, heart failure, stroke, TIA, diabetes and Parkinson’s were additionally controlled for. These variables were not controlled for in models of cause specific mortality due to high correlation of these conditions with deaths due to CVD. When modelling incident CVD, those with CVD at baseline were removed from analysis.

## Results

### Participants

Of the 4956 participants available, 4336 TILDA participants (mean age 61.5, 53.7% female) were included. During the follow-up time period, 9.7% (*n* = 422) were confirmed as deceased as of the 31st January 2022. Of these; 116 were due to deaths of the circulatory system, 198 were due to cancer and 108 other causes. Due to sample size limitations deaths due to respiratory illnesses were combined with other causes and so of the 108 mortalities classified as “other”, 31 are known to be due to respiratory illnesses.

### Descriptive analysis: univariate summary

Table 1 shows a summary of each of the covariates by mortality status. As can be seen, age, sex female, lower education, hypertension and depression/anxiety/psychosis were all positively associated with all-cause mortality. Only age and hypertension were found to differ according to mortality cause.

**Fig. 2:**
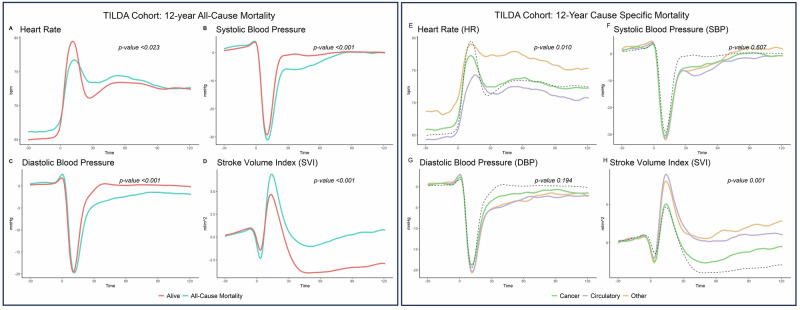
Mean haemodynamic responses by mortality status. Left Panel (**A**-**D**) Mean trace of haemodynamic signals according to All-Cause Mortality survival status for HR (**A**); SBP (**B**); DBP (**C**) and SVI (**D**). Time 0 denotes stand time. Right Panel (**E**-**H**) Mean trace of haemodynamic signals according to Cause-Specific Mortality status for HR (**E**); SBP (**F**); DBP (**G**) and SVI (**H**). Dashed black line indicates the mean curve for participants who were alive at the last day of follow up 31st January 2022 for reference. Time 0 denotes stand time. Reported *p*-values are the result of a functional ANOVA test using a permutation test on a basis function representation of the data (see Supplementary Tables 1 and 2 for full results).

Figure 2A-D shows the mean trace of each of the haemodynamic signals according to survival status for all-cause mortality. As can be seen, there is evidence to suggest that the mean trace of HR, SBP DBP and SVI differ according to survival status. Specifically, the mortality group have HR that is characterised by elevated supine baseline, a blunted peak on standing and a more gradual recovery between 10-40 seconds post standing (Fig. 2A). Recovery of both SBP and DBP in this group is muted between the post-stand nadir and ~70 seconds post standing (Fig. 2B, C). Change from baseline SVI is characterised by a higher post stand peak and elevated post-standing values in the recovery period from 30 seconds onwards in the mortality group (Fig. 2D).

**Table. 1:** Descriptive Summary of TILDA participants

Variable	Not Deceased (*n* = 3914)	Deceased All-Cause (*n* = 422)	*p*-value (All-Cause)^a^	Deceased Cause- Specific			
				Circulatory System (*n* = 116)	Cancer (*n* = 198)	Other (*n* = 108)	*p*-value Cause Specific^¥^
Age at baseline, mean (SD)	60.57 (7.57)	70.17 (8.90)	<0.001	71.80 (9.41)	68.21 (8.75)	72.02 (7.90)	<0.001
Sex Female, *n* (%)	2153 (55.0%)	177 (41.9%)	<0.001	46 (39.7%)	89 (44.9%)	42 (38.9%)	0.497
Education, *n* (%)							
Primary	767 (19.6%)	144 (34.1%)	<0.001	49 (42.2%)	64 (32.3%)	31 (28.7%)	0.262
Secondary	1677 (42.8%)	153 (36.3%)		37 (31.9%)	75 (37.9%)	41 (38.0%)	
Third	1470 (37.6%)	125 (29.6%)		30 (25.9%)	59 (29.8%)	36 (33.3%)	
BMI, mean (SD)	28.52 (4.85)	28.51 (5.08)	0.954	28.41 (4.96)	28.66 (5.02)	28.34 (5.36)	0.841
Hypertension, *n* (%)	2082 (53.2%)	314 (74.4%)	<0.001	99 (85.3%)	134 (67.7%)	81 (75.0%)	0.002
Depression/anxiety/ psychosis, *n* (%)	617 (15.8%)	89 (21.1%)	<0.009	19 (16.4%)	47 (23.7%)	23 (21.3%)	0.304
High Cholesterol, *n* (%)	3015 (77.0%)	327 (77.5%)	<0.989	95 (81.9%)	155 (78.3%)	77 (71.3%)	0.154
CVD and/or Diabetes, *n* (%)	479 (12.2%)	140 (33.2%)		51 (44%)	52 (26.3%)	37 (34.3%)	0.005

^a^*p*-value (All-Cause) for continuous variables is the result of an independent samples *t*-test which tests for a difference in the mean value between groups; for categorical variables this is the result of a Chi-Square test of difference in proportions.

^**¥**^*p*-value Cause Specific is the result of an ANOVA test for difference in mean values between circulatory, cancer and other causes of mortality for continuous variables. For categorical variables the *p*-value is the result of a Chi-Squared test between the three causes of death.

The *p*-value in each case is the result of a functional ANOVA analysis using a permutation test on a basis function representation of the data. As a sensitivity analysis, several types of ANOVA tests were conducted (with varying assumptions regarding the distribution of the data and structure of the covariance matrix) and found not to change the conclusions of the analysis (see Supplementary Tables 1 and 2 for full results).

Figure 2 (E-H) depict the mean trace of each of the haemodynamic signals according to mortality cause (see Supplementary Table 3 for *p*-values resulting from pairwise comparisons). As can be seen, only HR and SVI differ significantly between the three recorded causes of mortality (*p*-value 0.01 and 0.001 Fig. 2E, H respectively). Deaths of the circulatory system have a blunted HR peak and recovery to standing (Fig. 2E) as well as an elevated change from baseline SVI from post-standing peak onwards compared to deaths due to cancer (Fig. 2H). The haemodynamic response for cancer mortality was in general more similar to those who survived 12-year follow-up when compared to circulatory and other causes (Fig. 2 E-H). Other mortality was characterised by elevated supine baseline and post-stand HR, as well as a poorly defined peak HR and recovery (Fig. 2E). The SVI response for other mortality was similar to HR.

### Multivariable analysis of 12-year all-cause mortality

**Fig. 3:**
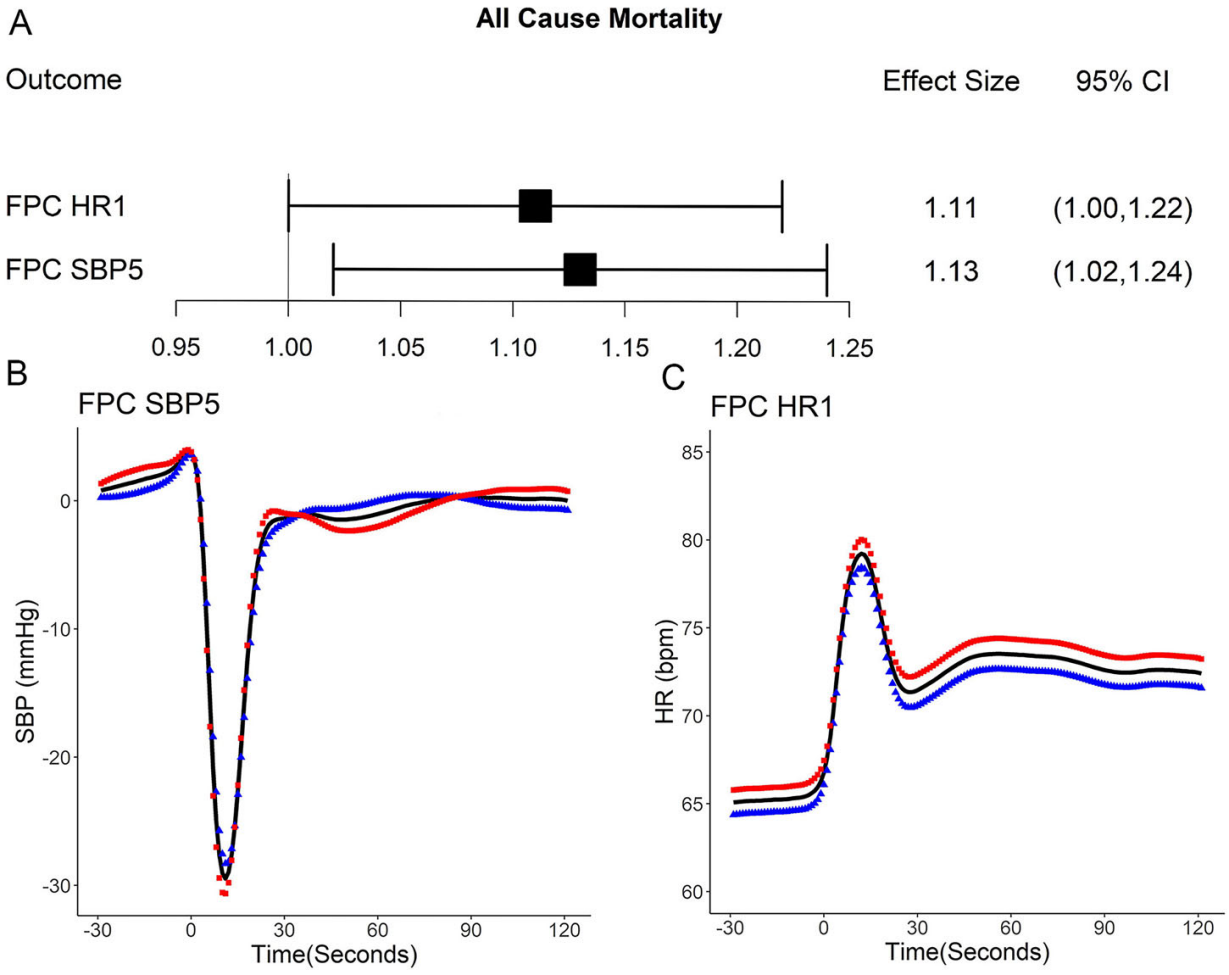
Associations with all-cause mortality. **A** Hazard ratio and 95% CI for the coefficients included in the model for all-cause mortality. **B** Modes of variation associated with all-cause mortality (mean signal +/- 30 x eigenfunction (principal component)). **B** Mode of variation for the 5th Systolic Blood Pressure principal component. **C** Mode of variation for the 1st Heart Rate principal component. Red squares represent how high scores on the indicated FPC affect the mean, blue triangles represent how low scores on the principal component affect the mean. The mean curve for each signal is indicated by the continuous black line.

Figure 3A shows the Hazard Ratio and 95% confidence intervals for the FPC scores associated with all-cause mortality (namely FPC HR1 and FPC SBP5 which refer to the first principal component for heart rate and the fifth component for SBP respectively). These Hazard Ratios are after adjustment for age; sex; education; smoking history; BMI; hypertension; high cholesterol, depression, anxiety or psychosis, frailty as well as a number of cardiovascular and neurogenerative diseases (see Methods section for the full set of covariates). For a list of all coefficients associated with mortality see Supplementary Table 4. Figure 3B depicts the mean HR signal (black solid) and how high versus low FPC scores for the first HR component (FPC HR1) perturb the mean signal. Figure 3C shows the equivalent information for the fifth SBP component (FPC SBP5). Figure 3B and C can be used to aid interpretation and understanding regarding which features of the FPC’s may be discriminating mortality status. To illustrate, the Hazard ratio for each standard deviation increase in FPC SBP5 is 1.13 (95% CI 1.02–1.24, *p* = 0.016) (Fig. 3A). Hence FPC SBP 5 is positively associated with all-cause mortality. High/positive scores on this component (red squares, Fig. 3B) discriminate higher baseline SBP and possibly blunted recovery of SBP from ~30–70 post stand. Therefore these features of SBP recovery may be driving the association with mortality. Similarly the scores related to component FPC.

HR1 were positively associated with mortality. As can be seen higher scores on this component are associated with elevated heart rate throughout the experiment (red squares, Fig. 3C).

### Multivariable analysis for cause-specific mortality

Supplementary Fig. 2A–D shows how the principal components associated with cause-specific mortality affect their respective mean curves. Supplementary Table 5 shows the HR 95% confidence intervals and *p*-values associated for the model on cause specific mortality. From Supplementary Table 5 it can be seen that FPC HR2 (the 2nd principal component for HR) was negatively associated with cardiovascular mortality (*p*-value 0.013) while the 5th component of SBP (FPC SBP5) was marginally positively associated (*p*-value 0.084). From Supplementary Fig. 2B, lower values on FPC HR2 (blue triangles) discriminate lower baseline and peak HR and higher HR from 30 seconds post stand onwards. Lower heart rate throughout, and in particular around the HR peak was indeed a feature of cardiovascular mortality (seen in Fig. 2 E).

Both FPC HR2 and FPC SVI1 were positively associated with cancer mortality. Respectively these discriminate a higher baseline HR, a higher and pronounced HR peak (red squares Supplementary Fig. 2B) and lower change from baseline SVI from the post stand maximum onwards (Supplementary Fig. 2D) which are also features of cancer mortality identified in Fig. 2 E and H.

**Fig. 4:**
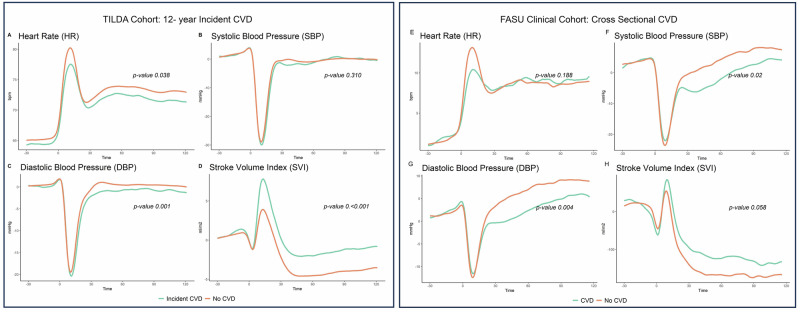
Mean haemodynamic responses by CVD status. Left Panel **A**-**D**: Baseline haemodynamic responses to standing for participants who had no history of CVD (wave1 2009-2010) for HR (**A**); SBP (**B**); DBP (**C**) and SVI (**D**). In all cases groups denote participants who subsequently went on to have incident CVD vs those who did not have CVD during 12-year follow-up. Right Panel **E**-**H** Mean haemodynamic responses to standing for patients with diagnosed CVD vs No CVD in a separate clinical cohort for HR (**E**); SBP (**F**); DBP (**G**) and SVI (**H**).

Other deaths were characterised by higher values on FPC HR1 and SBP5 along with lower values on FPCSV1 which is associated with impaired recovery of SPB from ~30–70 after stand; elevated SVI in the post stand recovery period as well as higher HR throughout the procedure (Fig. 4 A, C and D).

### Sensitivity analysis: beyond consensus measures

As SBP5 which discriminates high baseline SBP and poor recovery of SBP from 30–70 seconds post stand was associated with all-cause as well as cause specific mortality; we wanted to ensure that our FPC scores were not merely acting as surrogates for baseline SBP, HR and/or orthostatic hypotension. To investigate this, we performed a sensitivity analysis to examine whether our dynamic measures performed any better than using consensus definitions of orthostatic hypotension, baseline SBP and baseline HR. Hence the above models were repeated with baseline SBP, baseline HR and with binary indicators of orthostatic hypotension (OH) at 10 second intervals from 10 to 60 seconds after standing. Interestingly there was no association between OH and deaths of the circulatory system, deaths from cancer nor other deaths. Regarding all-cause mortality OH40 was significantly associated, however none of the other BP measurements had any association. Baseline HR was only associated with cardiovascular deaths but not with all-cause mortality nor cancer nor other causes of death. Baseline SBP was not predictive of all-cause nor cause specific mortalities (see Supplementary Table 6 for full results).

### Model accuracy

The area under the ROC curve (AUC) as a function of age at death for all-cause and cause specific mortality are depicted in Supplementary Fig. 3. In all cases the cross-validated AUC is based on 20% test data which was not used to train the model. The AUC for all-cause mortality was quite stable across age and ranged from 91.2% (age 67) to 75.9% (age 87). With respect to circulatory deaths, the AUC more accurately discriminated mortality at younger ages (from 65–71) than those at older ages. There was good discrimination of cancer mortalities from ages 65–83 was (average AUC 86.47%) with wider confidence intervals beyond that age range while other mortalities could not be accurately discriminated (95% CI overlapped 50% in nearly all cases). The improved predictive accuracy for cancer mortality may perhaps be due to this being the most common cause of death in our sample and hence the highest powered (46.9% of deaths compared to 27.5% due to deaths of the circulatory system and 25.6% due to other causes).

### Investigating 12-year incident CVD

Figure 4 shows the mean baseline haemodynamic profiles of participants with 12-year incident CVD (*n* = 229) versus those who did not have CVD after 12-year follow up (*n* = 2182). Those with a history of CVD at baseline were removed from the analysis. Supplementary Table 7 shows the results of the functional ANOVA tests conducted. As can be seen HR, DBP and SV were significantly associated with 12-year incident CVD status in univariate analysis (Figures A, C, D, *p*-value 0.038,0.001 and <0.001 respectively). Those with incident CVD had on average lower HR throughout the experiment and in particular a blunted recovery to standing around the HR peak, followed by lower HR in the recovery from 30-180 seconds after standing. They also had a significantly lower DBP/blunted recovery of DBP on standing in the recovery period from ~30 seconds after stand onwards as well as an elevated change from baseline SVI from the initial response to standing ~5 seconds after standing to the end of the experiment.

With regards to multivariable analysis; after adjusting for age, sex, education, medication usage, BMI, smoking and frailty status, the component scores FPC HR1 and FPC SV1 remained significantly associated with incident CVD status.

The components associated with incident CVD were related to lower HR and elevated post stand SVI which was in line with the univariate analysis depicted in Fig. 4 (see Supplementary Fig. 4 for mean trace and how high versus low scores on the FPCs significantly associated with incident CVD perturb this mean curve. See also Supplementary Table 8 for the odds ratio and 95% CI of variables significantly associated with incident CVD). Supplementary Fig. 6 shows the eigenfunctions associated with all outcomes and their respective variance explained.

Regarding model accuracy, the mean AUC based on 20% test data was 68.9%, 95% CI (62.6%,73.5%); sensitivity 61.2% and specificity 65.6%.

A recap and summary of the FPC’s their defining characteristics and the outcomes they are associated with can be found in Table 2.

**Table. 2:** Summary of the main results

FPC	Pattern	Outcome associated with
HR1	Elevated HR at pre and post stand	All-cause mortality Other Mortality Incident CVD
HR2 (positive association)	Higher baseline HR and pronounced peak HR post standing. Lower HR from 30 seconds post-stand onwards.	Circulatory System mortality
HR2 (negative association)	Lower baseline HR and impaired/lower peak HR post standing. Higher HR from 30 seconds post-stand onwards.	Cancer mortality
SBP5	High baseline supine SBP and poor recovery of SBP from 30-70 seconds post-stand	All-cause mortality Other Mortality
SV1 (negative association)	Elevated SVI throughout but more pronounced elevation from 30 seconds post stand onwards.	Incident CVD Other mortality
SVI (positive association)	Lower SVI throughout but more pronounced lowering of SVI from 30 seconds post stand onwards.	Cancer mortality

### External validation on a clinical cohort

The FPC models trained on the TILDA study were exported and validated on a separate clinical cohort (*n* = 347; 56.2% female; age mean (sd) 70.4 (10.3)) from the Falls and Syncope Unit in St James’ Hospital. Of the 376 total patients 167 (48.1%) had CVD. Figure 4 E-H shows the mean haemodynamic curves for those with CVD vs no CVD for this cohort.

After adjustment for age, sex, BMI, smoking history, and medication usage the second FPC for diastolic blood pressure which discriminates a blunted drop in blood pressure to the nadir and impaired blood pressure recovery from 35 seconds onwards remained significantly associated with presence of cardiovascular disease (see Supplementary Fig. 5). The AUC on the 20% test data was 0.64 95% CI (0.56,0.71), sensitivity 0.63, specificity 0.60.

## Discussion

In this study of over 4300 community dwelling older adults, we have shown that functional principal component scores of haemodynamic responses to standing were independently associated with 12 year all-cause and cause specific mortality and also independently associated with 12-year incident CVD even after robust adjustment for health, frailty, behavioural and demographic risk factors (average cross validated AUC 83.7%,81.8% and 70.0% for all-cause, cancer and circulatory system mortality respectively; AUC 68.9% for incident CVD). We also showed that recovery of DBP was associated with cross sectional CVD in a separate clinical cohort. Although AS has previously been associated with all-cause mortality and CVD^11–17,26–32^, to the author’s knowledge this is the first time that AS has been associated with cause-specific mortality and also the first time that wider signs of autonomic dysfunction beyond simple measures of OH are associated with incident CVD and indeed are detectable up to 12 years prior to a CVD event.

Briefly, all-cause mortality was associated with principal components which discriminated elevated baseline SBP and impaired SBP recovery from ~30–60 seconds after standing as well as higher HR in general. Mortality of the circulatory system was associated with principal components of HR which discriminated lower supine baseline HR and blunted HR peak as well as impaired recovery of SBP ~ 30–70 seconds after stand. Cancer mortality was associated with components which discriminated lower change from baseline SVI from ~10 seconds post stand onwards and a pronounced/well defined HR peak. These characteristics of cancer mortality were more similar to those who survived than to those who died of CVD and other causes. Similar to circulatory system mortality, other mortalities were also associated with components which discriminated impaired SBP recovery as well as elevated HR and SVI in recovery to standing. Meanwhile, incident CVD was associated with lower HR throughout and an elevated SVI in the initial and sustained response to standing from ~5 seconds post stand to the end of the experiment.

Many of these findings are in line with previous research. In particular^47^, discovered that slower speed of heart rate recovery from 10-20 seconds after standing was associated with all-cause mortality and likely reflected dysregulation of the response of the parasympathetic nervous system; as parasympathetic inhibition in the immediate response to standing and subsequent reactivation in the initial recovery ~10 seconds after standing are thought to be responsible for the peak and subsequent drop in HR in response to orthostasis. The work of^62^ and others also found that higher resting heart rate was positively associated with mortality (see also refs. ^49,50^).

Supine hypertension either on its own or with concomitant OH has previously been associated with shorter survival and higher risk of cardiovascular events as it represents a dysfunctional response in the autonomic reflexes required to maintain blood pressure upon standing^63,64^. Other studies have found evidence of U shaped association with respect to supine blood pressure and age where lower supine SBP was associated with mortality in those aged 85+^65^. Interestingly, our results showed that neither OH from 10–60 seconds post stand nor resting supine SBP were significantly associated with 12-year all cause nor cause specific mortality (the only exception being OH40 for all cause-mortality). However, the FPC scores for the 5th SBP component which discriminated higher supine SBP and impaired recovery of SBP between ~30–70 seconds after stand were significantly associated with all-cause, circulatory system and other mortalities in multivariable analyses. One reason for this may be that the FPC scores allow for information across the time-varying haemodynamic curves to be incorporated into the model which may constitute a much richer and flexible source of information than a dichotomous or scalar summary taken at individual timepoints. Such information may also allow for subclinical features of autonomic dysregulation to be detected which may otherwise be missed by focusing on simplistic summaries such as the classical measurement of OH60.

Interestingly, despite elevated HR being a well-known risk factor for both CVD and mortality, those who were otherwise healthy at baseline but developed CVD in the intervening 12 years had lower baseline HR throughout the active stand procedure including at supine rest. However, the relative difference between those who remain CVD free and those with incident CVD is most pronounced around the initial response at the heart rate peak ~10–20 after standing and so again may be an early indicator of autonomic dysregulation in particular over activation of the sympathetic nervous system^47^.

The predominant features of autonomic dysregulation found in otherwise healthy adults who go on to have incident CVD are strikingly similar to the validation cohort of those who already have CVD from the validation clinical cohort of patients referred to the Falls and Syncope Unit i.e. blunted HR peak, impaired BP recovery from ~20 seconds after stand and elevated SVI from the stand point onwards. The fact that these features are present in those with incident CVD and also present but more pronounced in those already with a diagnosis of CVD suggests that autonomic dysregulation and the haemodynamic response to a stressor such as standing may be an important early indicator of future CVD.

We have shown that our FPCA model which was trained on a relatively healthy population representative sample of Irish adults aged 50 and over can be easily exported and used in other settings. Also, the fact that the component capturing impairment of DBP recovery to standing was significantly associated with CVD in a cohort of clinical patients with many more underlying conditions than typical in the training cohort showcases the flexibility and robustness of such a modelling approach. Using CVD as an example outcome in this case, we have demonstrated how our principal component scores, trained using TILDA data, can easily be constructed for any clinical setting which performs the active stand and also used to model any other clinical outcome of interest such as cognitive impairment or future falls.

One of the main limitations of this study is that the subsample of participants who partook in the TILDA health assessment were a much healthier subsample of the larger study and therefore the effects of peripheral haemodynamic responses to orthostasis on mortality may be underestimated in some cases. Further bias regarding study attrition is not present here as for all but *n* = 29 participants their mortality status is known exactly as of the 31st January 2022 due to external data linkage with the national death registry regardless of attrition status from the TILDA study. 12-year incident CVD was investigated on the TILDA cohort in the absence of a fully randomised cohort to exclude as much as possible the risk of reverse causality. The possibility of survivor bias affecting these results was also taken into consideration as much as possible by validating the association of the FPC scores on a clinical cohort with existing disease and very different baseline characteristics. The fact that our finding persisted in both 12-year incident as well as existing CVD and across two very different cohorts adds weight to the generalisability and credence of these findings.

The FPC scores provide a small set of simple continuous measurements which capture information regarding complex trends in high dimensional haemodynamic profiles and we have demonstrated how they can easily be calculated automatically in a clinical setting and incorporated into prediction models for prediction of health outcomes. Our findings showed that the FPC scores were associated with all-cause as well as cause specific mortality in cases where traditional measurements such as absolute BP, HR and OH were not which shows the additional benefit of this method above and beyond traditional measurements of autonomic dysfunction. Furthermore, to our knowledge no study to date has shown that AS has been associated with cause-specific mortality and nor that wider signs of autonomic dysfunction beyond simple measures of absolute BP, HR and OH are associated with incident CVD and indeed are detectable up to 12 years prior to a CVD event.

## Conclusions

This study has shown that functional principal component analysis can be used to uncover dynamic features of hemodynamic responses to standing. Our models accurately discriminated 12-year incident CVD as well as 12-year all-cause and cause specific mortality in a population cohort of adults aged 50 + . We have also demonstrated how these models can be incorporated into a clinical setting and used for prediction of health outcomes, identifying impaired recovery of DBP as a significant factor associated with CVD after covariate adjustment.

To our knowledge this is the first study to incorporate dynamic data driven information over the entire trace of the haemodynamic response to standing to investigate associations with prevalent and incident CVD and mortality. The fact that components which discriminated high BP and impaired recovery of BP were associated with 12-year mortality when traditional summary measures of OH and baseline BP were not; suggests that incorporating information across the entire response to standing allows for richer and more flexible associations with health outcomes to be uncovered and allows for a better understanding of the underlying physiology leading to such associations.

## Supplementary information

Supplementary material

Supplementary Data 1

Supplementary Data 2

Supplemtary Data 3

## Data Availability

The dataset(s) supporting the conclusions of this article are not publicly available due to data protection regulations but are accessible at TILDA on reasonable request. The procedures to gain access to TILDA data are specified at https://tilda.tcd.ie/data/accessing-data/. The source data for Fig. 2 is in Supplementary Data 1; source data for Fig. 3 is in Supplementary Data 2; source data for Fig. 4 is in Supplementary Data 3.

## Code availability

All code supporting the conclusions of this article are available on request. Please email tilda@tcd.ie.
