# Supplementary material for: Responses to active stand test predict 12-year incident cardiovascular disease and mortality
Source: Commun Med (Lond). 2025 Dec 13;6:4. doi: 10.1038/s43856-025-01253-3 (PMC12764857; doi:10.1038/s43856-025-01253-3)
Supplement: Supplementary file 1 — Supplementary material [file 43856_2025_1253_MOESM1_ESM.docx]

**Supplementary Methods 1: Functional ANOVA**

For univariate summaries of the peripheral haemodynamic signals, group means were compared using functional ANOVA. Briefly, for each of the haemodynamic signals considered the participants’ response curves are represented by $X_{hj}\left( t \right)$, where $t=1, \ldots,151$ are the timepoints at which data is captured for each individual (once per second); $h=1, \ldots,n_{l}$ indexes over the number of groups being compared (i.e. $l=3$ in the case of cause specific mortality); $j=1, \ldots,n_{h}$ indexes over individuals in group $i$. The functional ANOVA tests the null hypothesis that group means $\mu_{h}$ are equal at each timepoint $t$ versus the alternative that at least one group mean is different.

$$H_{o}: \mu_{1}\left( t \right)=\ldots=\mu_{l}(t)$$

To ensure that results and conclusions drawn are not dependent on the underlying assumptions regarding any one version of the functional ANOVA test, multiple versions of this test all with varying assumptions were performed and the results for each reported (Tables S1,S2). The conclusions of the analysis were not affected by varying test assumptions.

**Supplementary Methods 2: Functional Principal Components Analysis (FPCA)**

FPCA is an extension of the well-known dimensionality reduction technique principal components analysis (PCA) for functional data (in this case haemodynamic responses as a function of time). In standard PCA an eigen decomposition is performed on the covariance matrix of multivariate data which results in eigenvalues and their accompanying eigenvectors commonly referred to as principal components. Eigenvalues represent the proportion of variation explained by each of the principal components. The principal components or eigenvectors themselves describe the direction of variation in the data. FPCA is a very similar except instead of eigenvectors associated with each eigenvalue; there are eigenfunctions associated with each eigenvalue which explain the orientation and scale of the variation in the data respectively. In a similar vein, as with PC scores in standard PCA where observations are projected onto the PC sub-space and their coordinates extracted; FPC scores can also be extracted for each individual and each component in FPCA as follows:

$$c_{ik}=\int\phi_{k}(t)[X_{i}(t)-\mu(t)]$$

Here $c_{ik}$are the component scores, $\phi_{k}$ refers to the $k^{th}$ eigenfunction and $X_{i}(t)$ refers to the response curve for observation $i$ at time $t$. These scores can then be input as coefficients into other standard multivariable models (58-60).

**Supplementary Methods 3: Elastic Net Regression**

To model incident CVD an elastic net logistic regression was implemented. As high class-imbalance was present for incident CVD (9.6% of participants had incident CVD); the model was trained across 1,000 balanced bootstrap samples to ensure that the associations discovered were not due to bias in selecting the control cases. Over each of the 1,000 permutated datasets we employed an elastic net penalised regression which is a common method for variable selection. The optimal $\lambda$ was chosen using 10-fold cross validation on the training data and $\alpha=0.5$. The variables which had non-zero coefficients in more than 50% of the permutations were considered consistently associated with CVD and presented in the results. Again, all performance metrics were calculated based on the 20% test set which was not used in any part of the model building.

**Supplementary Tables:**

**Supplementary Table 1: Results for the full list of ANOVA tests performed for each of the haemodynamic signals on survival status for All-Cause mortality.**

| *Test Type* | *Heart Rate p-value* | *Systolic Blood Pressure p-value* | *Diastolic Blood Pressure p-value* | *Stroke Volume Index p-value* |
| --- | --- | --- | --- | --- |
| FP Test: Permutation test based on a basis function representation | 0.023 | <0.001 | <0.001 | <0.001 |
| CH Test: L2-norm-based parametric bootstrap test for homoscedastic samples | 0.026 | <0.001 | <0.001 | <0.001 |
| CS Test: L2-norm-based parametric bootstrap test for heteroscedastic samples | 0.050 | <0.001 | <0.001 | <0.001 |
| L2-norm-based test with naïve method of estimation | 0.025 | <0.001 | <0.001 | <0.001 |
| L2-norm-based test with bias-reduced method of estimation | 0.025 | <0.001 | <0.001 | <0.001 |
| L2 norm based on bootstrap test | 0.052 | <0.001 | <0.001 | <0.001 |
| F-type test with naïve method of estimation | 0.025 | <0.001 | <0.001 | <0.001 |
| F-type test with bias-reduced method of estimation | 0.025 | <0.001 | <0.001 | <0.001 |
| F-type bootstrap test | 0.044 | <0.001 | <0.001 | <0.001 |
| Globalizing Pointwise F-test | 0.027 | <0.001 | <0.001 | <0.001 |
| Fmax bootstrap test | <0.001 | <0.001 | <0.001 | <0.001 |

**Supplementary Table 2: Results for the full list of ANOVA tests performed for each of the haemodynamic signals on cause-specific mortality.**

| *Test Type* | *Heart Rate p-value* | *Systolic Blood Pressure p-value* | *Diastolic Blood Pressure p-value* | *Stroke Volume Index p-value* | *Cardiac Index p-value* |
| --- | --- | --- | --- | --- | --- |
| FP Test: Permutation test based on a basis function representation | 0.01 | 0.607 | 0.194 | 0.001 | 0.001 |
| CH Test: L2-norm-based parametric bootstrap test for homoscedastic samples | 0.010 | 0.608 | 0.213 | 0.006 | 0.003 |
| CS Test: L2-norm-based parametric bootstrap test for heteroscedastic samples | 0.007 | 0.624 | 0.204 | 0.006 | 0.002 |
| L2-norm-based test with naïve method of estimation | 0.005 | 0.626 | 0.207 | <0.001 | <0.001 |
| L2-norm-based test with bias-reduced method of estimation | 0.005 | 0.626 | 0.206 | <0.001 | <0.001 |
| L2 norm based bootstrap test | 0.007 | 0.639 | 0.199 | 0.003 | <0.001 |
| F-type test with naïve method of estimation | 0.006 | 0.626 | 0.208 | 0.001 | <0.001 |
| F-type test with bias-reduced method of estimation | 0.006 | 0.627 | 0.208 | 0.001 | <0.001 |
| F-type bootstrap test | 0.007 | 0.634 | 0.193 | 0.003 | <0.001 |
| Globalizing Pointwise F-test | 0.006 | 0.687 | 0.263 | 0.001 | <0.001 |
| Fmax bootstrap test | 0.006 | 0.931 | 0.233 | 0.009 | 0.003 |

**Supplementary** **Table 3 P-values resulting from a pairwise comparison of mortality cause.**

| Pairwise Comparison | p-value |
| --- | --- |
| *Heart Rate* |  |
| Cancer Vs Circulatory | 0.222 |
| Cancer Vs Other | 0.022 |
| Circulatory Vs Other | 0.002 |
| *Systolic Blood Pressure* |  |
| Cancer Vs Circulatory | 0.564 |
| Cancer Vs Other | 0.536 |
| Circulatory Vs Other | 0.445 |
| *Diastolic Blood Pressure* |  |
| Cancer Vs Circulatory | 0.131 |
| Cancer Vs Other | 0.122 |
| Circulatory Vs Other | 0.971 |
| *Stroke Volume Index* |  |
| Cancer Vs Circulatory | 0.008 |
| Cancer Vs Other | 0.004 |
| Circulatory Vs Other | 0.545 |

**Supplementary Table 4 Hazard Ratio, 95% CI and p-value for variables included in the final model of all-cause mortality after applying step-wise variable selection**

|  | Hazard Ratio | LCI | UCI | p-value |
| --- | --- | --- | --- | --- |
| Age | 0.90 | 0.88 | 0.92 | <0.001 |
| Female | 0.64 | 0.51 | 0.80 | <0.001 |
| Education Secondary | 0.86 | 0.67 | 1.10 | 0.238 |
| Education Third | 0.68 | 0.51 | 0.90 | 0.007 |
| Depression/Anxiety/Psychosis | 1.34 | 1.01 | 1.76 | 0.040 |
| Former Smoker | 1.44 | 1.13 | 1.84 | 0.004 |
| Current Smoker | 2.76 | 2.02 | 3.79 | <0.001 |
| Pre-Frail | 1.19 | 0.83 | 1.54 | 0.171 |
| Frail | 2.08 | 1.54 | 2.81 | <0.001 |
| FPC HR 1 | 1.08 | 0.98 | 1.20 | 0.109 |
| FPC SBP 5 | 1.14 | 1.04 | 1.26 | 0.007 |

**Supplementary Table 5: Coefficients included in the final model for cause specific mortality using competing risks analysis and stepwise variable selection:**

| Model | HR | HR LCI | HR UCI | p-value |
| --- | --- | --- | --- | --- |
| *Circulatory System* |  |  |  |  |
| Age | 0.96 | 0.94 | 0.98 | <0.001 |
| Sex Female | 0.65 | 0.44 | 0.98 | 0.038 |
| Education |  |  |  |  |
| Secondary | 0.65 | 0.40 | 1.03 | 0.069 |
| Third | 0.58 | 0.34 | 0.97 | 0.039 |
| Hypertension | 2.00 | 1.10 | 3.62 | 0.023 |
| Frailty Index |  |  |  |  |
| Pre-Frail | 1.02 | 0.61 | 1.72 | 0.931 |
| Frail | 2.44 | 1.46 | 4.07 | <0.001 |
| FPC HR2 | 0.78 | 0.65 | 0.95 | 0.013 |
| FPC SBP5 | 1.15 | 0.98 | 1.35 | 0.084 |
| *Cancer* |  |  |  |  |
| Age | 0.96 | 0.94 | 0.97 | <0.001 |
| Depression/Anxiety/Psychosis | 1.49 | 1.03 | 2.18 | 0.037 |
| Smoking Status |  |  |  |  |
| Former Smoker | 1.54 | 1.08 | 2.20 | 0.037 |
| Current Smoker | 2.75 | 1.77 | 4.27 | <0.001 |
| FPC HR2 | 1.19 | 1.01 | 1.40 | 0.037 |
| FPC SV1 | 1.19 | 1.02 | 1.39 | 0.025 |
| FPC SV2 | 0.86 | 0.71 | 1.04 | 0.112 |
| *Other* |  |  |  |  |
| Depression/Anxiety/Psychosis | 1.56 | 0.95 | 2.56 | 0.080 |
| Smoking Status |  |  |  |  |
| Former Smoker | 2.06 | 1.26 | 3.36 | 0.004 |
| Current Smoker | 2.71 | 1.41 | 5.23 | 0.003 |
| FPC HR1 | 1.48 | 1.22 | 1.80 | <0.001 |
| FPC SBP5 | 1.28 | 1.07 | 1.53 | 0.007 |
| FPC SVI1 | 0.68 | 0.55 | 0.85 | <0.001 |

**Supplementary Table 6: Association between orthostatic hypotension at 10 second intervals post-stand and mortality.**

| Model | HR | HR LCI | HR UCI | p-value |
| --- | --- | --- | --- | --- |
| *All-Cause Mortality* |  |  |  |  |
| Baseline absolute HR | 1.00 | 1.00 | 1.00 | 0.463 |
| Baseline absolute SBP | 1.00 | 1.00 | 1.01 | 0.088 |
| OH 10 | 1.06 | 0.77 | 1.46 | 0.713 |
| OH 20 | 1.05 | 0.84 | 1.31 | 0.678 |
| OH 30 | 1.23 | 0.97 | 1.57 | 0.087 |
| OH 40 | 1.36 | 1.06 | 1.75 | 0.016 |
| OH 50 | 1.14 | 0.87 | 1.49 | 0.330 |
| OH 60 | 1.19 | 0.90 | 1.57 | 0.232 |
| *Circulatory System* |  |  |  |  |
| Baseline absolute HR | 1.00 | 0.99 | 1.00 | 0.003 |
| Baseline absolute SBP | 1.00 | 1.00 | 1.014 | 0.280 |
| OH 10 | 1.00 | 0.56 | 1.79 | 0.990 |
| OH 20 | 1.02 | 0.68 | 1.53 | 0.940 |
| OH 30 | 1.31 | 0.83 | 2.08 | 0.250 |
| OH 40 | 1.40 | 0.88 | 2.24 | 0.160 |
| OH 50 | 1.05 | 0.63 | 1.73 | 0.870 |
| OH 60 | 0.90 | 0.51 | 1.56 | 0.700 |
| *Cancer* |  |  |  |  |
| Baseline absolute HR | 1.01 | 1.00 | 1.02 | 0.250 |
| Baseline absolute SBP | 1.00 | 1.00 | 1.01 | 0.630 |
| OH 10 | 0.88 | 0.57 | 1.37 | 0.570 |
| OH 20 | 1.00 | 0.72 | 1.39 | 0.990 |
| OH 30 | 0.99 | 0.69 | 1.42 | 0.940 |
| OH 40 | 1.04 | 0.71 | 1.54 | 0.840 |
| OH 50 | 0.98 | 0.65 | 1.49 | 0.930 |
| OH 60 | 1.28 | 0.85 | 1.93 | 0.230 |
| *Other* |  |  |  |  |
| Baseline absolute HR | 1.00 | 0.99 | 1.01 | 0.94 |
| Baseline absolute SBP | 1.00 | 0.99 | 1.02 | 0.470 |
| OH 10 | 1.59 | 0.77 | 3.29 | 0.210 |
| OH 20 | 1.10 | 0.70 | 1.73 | 0.680 |
| OH 30 | 1.42 | 0.88 | 2.27 | 0.140 |
| OH 40 | 1.49 | 0.90 | 2.45 | 0.120 |
| OH 50 | 1.34 | 0.82 | 2.19 | 0.250 |
| OH 60 | 1.24 | 0.72 | 2.13 | 0.440 |

**Supplementary Table 7 Results for the full list of ANOVA tests performed for each of the haemodynamic signals on incident CVD.**

| Test Type | Heart Rate p-value | Systolic Blood Pressure p-value | Diastolic Blood Pressure p-value | Stroke Volume Index p-value |
| --- | --- | --- | --- | --- |
| FP Test: Permutation test based on a basis function representation | 0.038 | 0.310 | 0.001 | <0.001 |
| CH Test: L2-norm-based parametric bootstrap test for homoscedastic samples | 0.046 | 0.268 | <0.001 | <0.001 |
| CS Test: L2-norm-based parametric bootstrap test for heteroscedastic samples | 0.057 | 0.341 | 0.003 | <0.001 |
| L2-norm-based test with naïve method of estimation | 0.047 | 0.310 | <0.001 | <0.001 |
| L2-norm-based test with bias-reduced method of estimation | 0.047 | 0.310 | <0.001 | <0.001 |
| L2 norm based bootstrap test | 0.060 | 0.331 | <0.002 | <0.001 |
| F-type test with naïve method of estimation | 0.047 | 0.311 | <0.001 | <0.001 |
| F-type test with bias-reduced method of estimation | 0.047 | 0.311 | <0.001 | <0.001 |
| F-type bootstrap test | 0.056 | 0.344 | 0.002 | <0.001 |
| Globalizing Pointwise F-test | 0.050 | 0.469 | <0.001 | <0.001 |
| Fmax bootstrap test | 0.003 | 0.070 | <0.001 | <0.001 |

**Supplementary Table 8 Mean Coefficients (Odds Ratio) along with their 2.5 and 97.5 percentiles from elastic net model of incident CVD. Variables with non-zero coefficients in at least 50% of the 1,000 bootstrap samples and whose association was consistent (2.5 and 97.5 percentile intervals did not overlap 1) were considered associated with the outcome and shown below. Note: covariates with zero coefficients over more than 50% of the bootstrap samples included: education, high cholesterol, depression/anxiety/psychosis, FPC HR1, FPC HR2, FPCs SV3-4 and FPCs DBP1-6.**

| Coefficient | OR (95% CI) |
| --- | --- |
| FPC SV1 | 0.88 (0.71, 0.995) |
| FPC HR1 | 0.89 (0.71, 0.997) |
| Age | 1.37 (1.11, 1.73) |
| Sex (Female) | 0.75 (0.48, 0.98) |
| Hypertension | 1.61 (1.08, 2.51) |
| Pre-Frail | 1.32 (1.01, 2.04) |
| Frail | 1.52 (1.01, 3.39) |

**Supplementary Figures:**


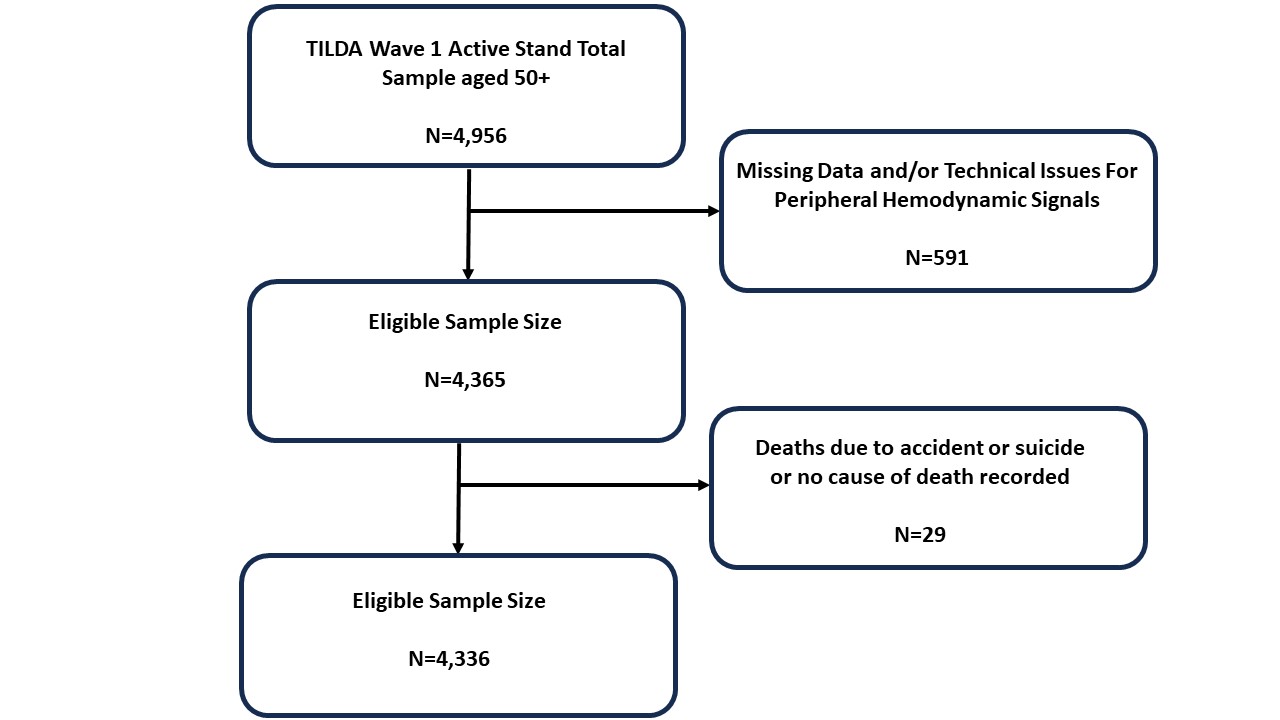


Supplementary Figure 1 Flow chart depicting exclusion criteria and eligible sample size included in final analysis.

Figure Title Flow Chart

**
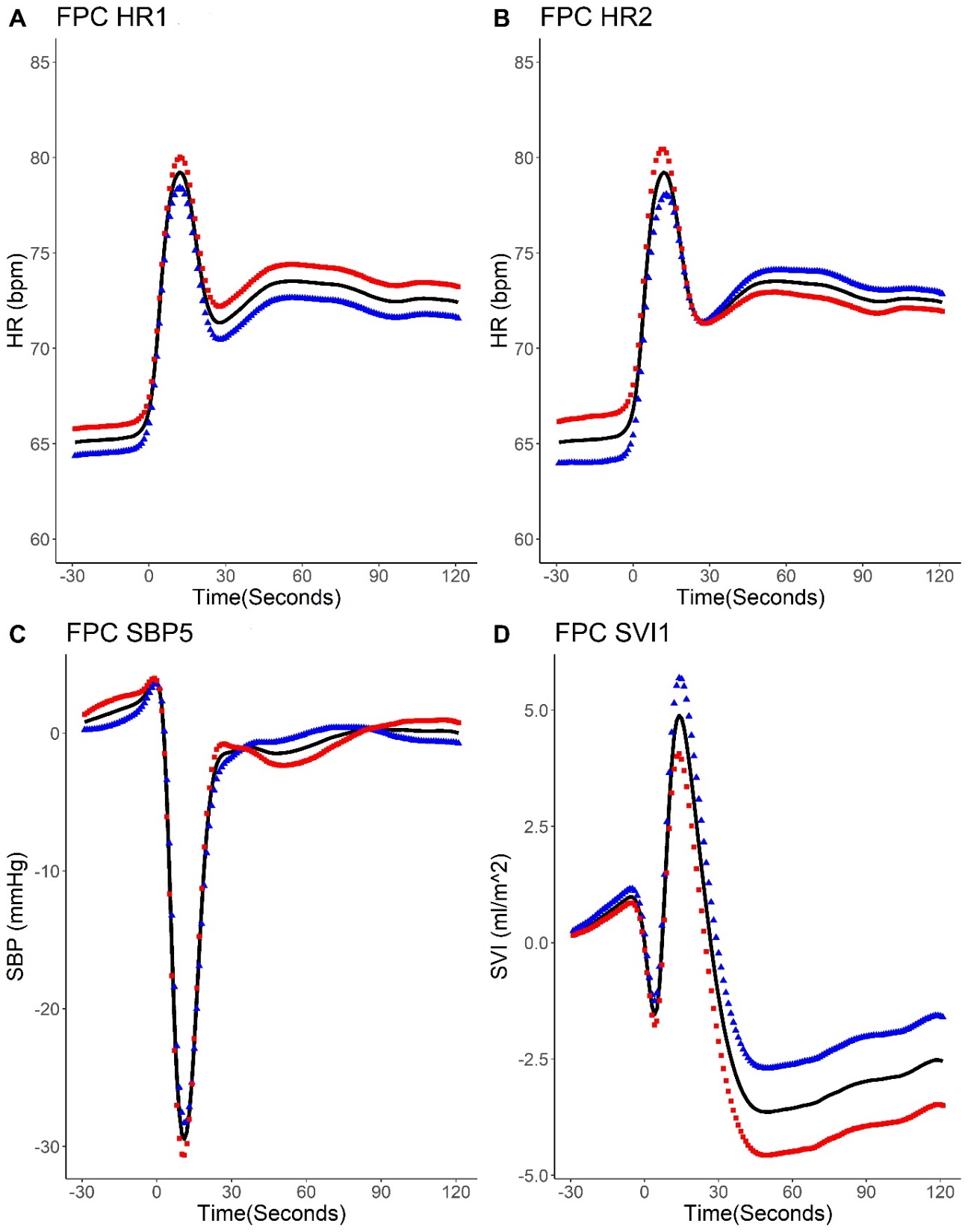
**

Supplementary Figure 2: How positive vs negative scores on the principal components associated with cause specific mortality impact the mean curve (Figure 2A relates to FPC HR1; Figure 2B to FPC HR2; Figure 2C to FPC SBP5 and Figure 2D to FPC SV1)). Red squares in each case represent how high (positive) scores on the indicated component affect the mean, blue triangles represent how low (negative) scores on the component affect the mean.

Figure Title: Associations with Cause-Specific Mortality


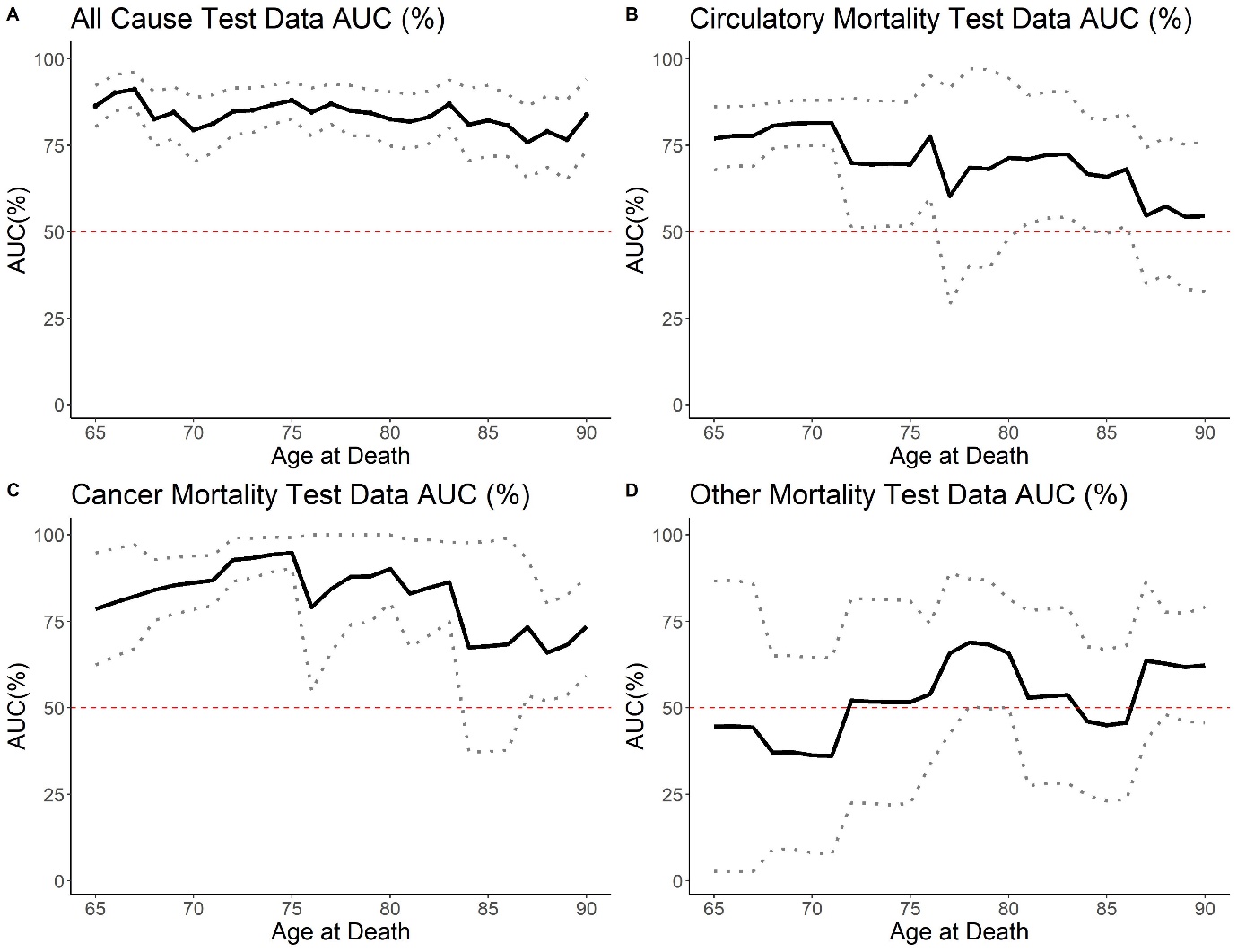


Supplementary Figure 3: Estimated time-dependent AUC and 95% CI according to age at death on 20% hold-out test data (A) All-Cause Mortality, (B) Circulatory Mortality, (C) Cancer Mortality and (D) Other Mortality.

Figure Title: Accuracy of Mortality Models


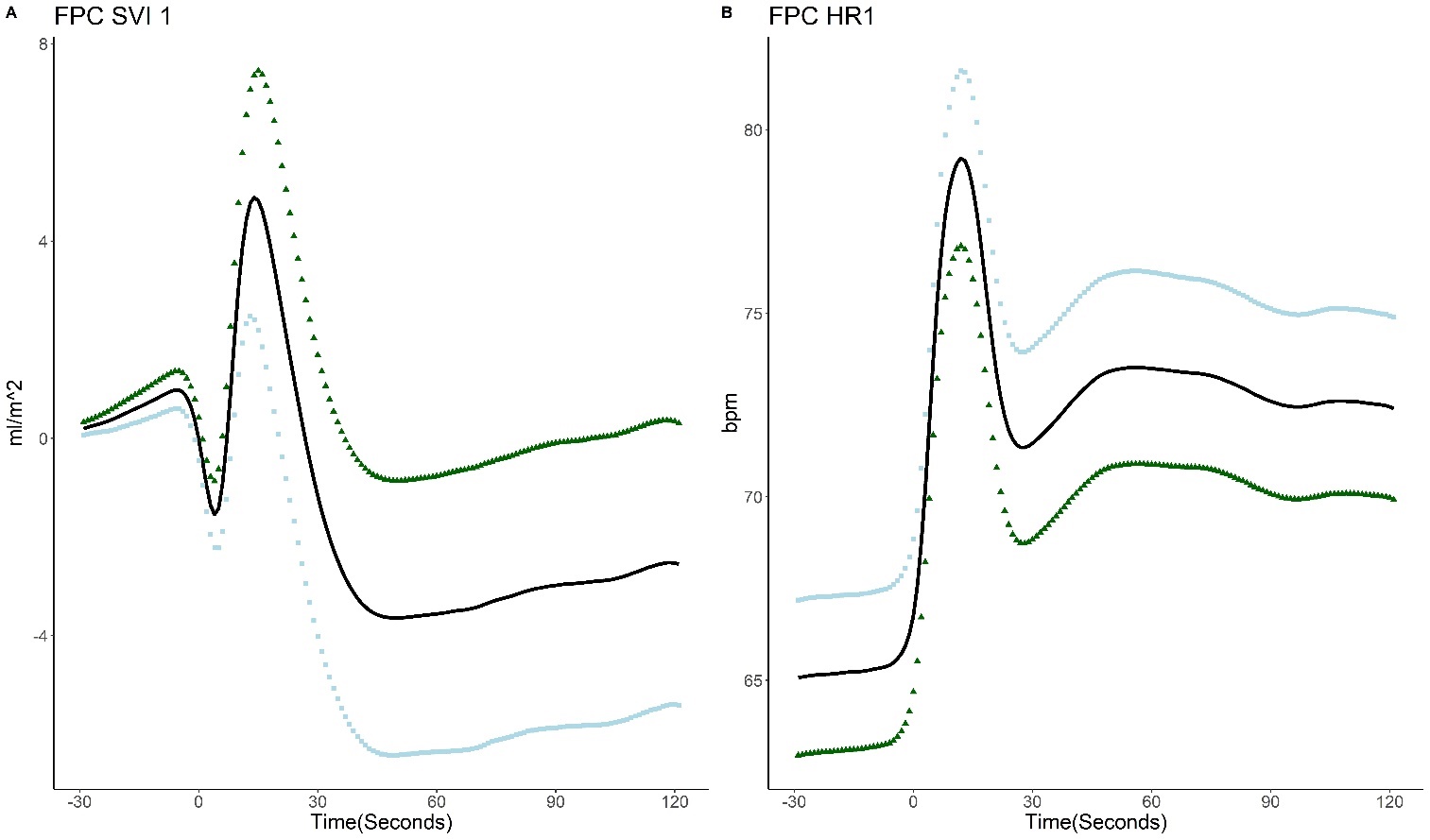


Supplementary Figure 4 (A) Mean trace (black) with FPCs added and subtracted from the mean for FPC SVI 1. (B) Mean trace (black) with FPCs added and subtracted from the mean for FPC HR 1. The direction of the principal component scores associated with incident CVD is represented by green triangles in each case.

Figure Title: Association with 12-year incident CVD


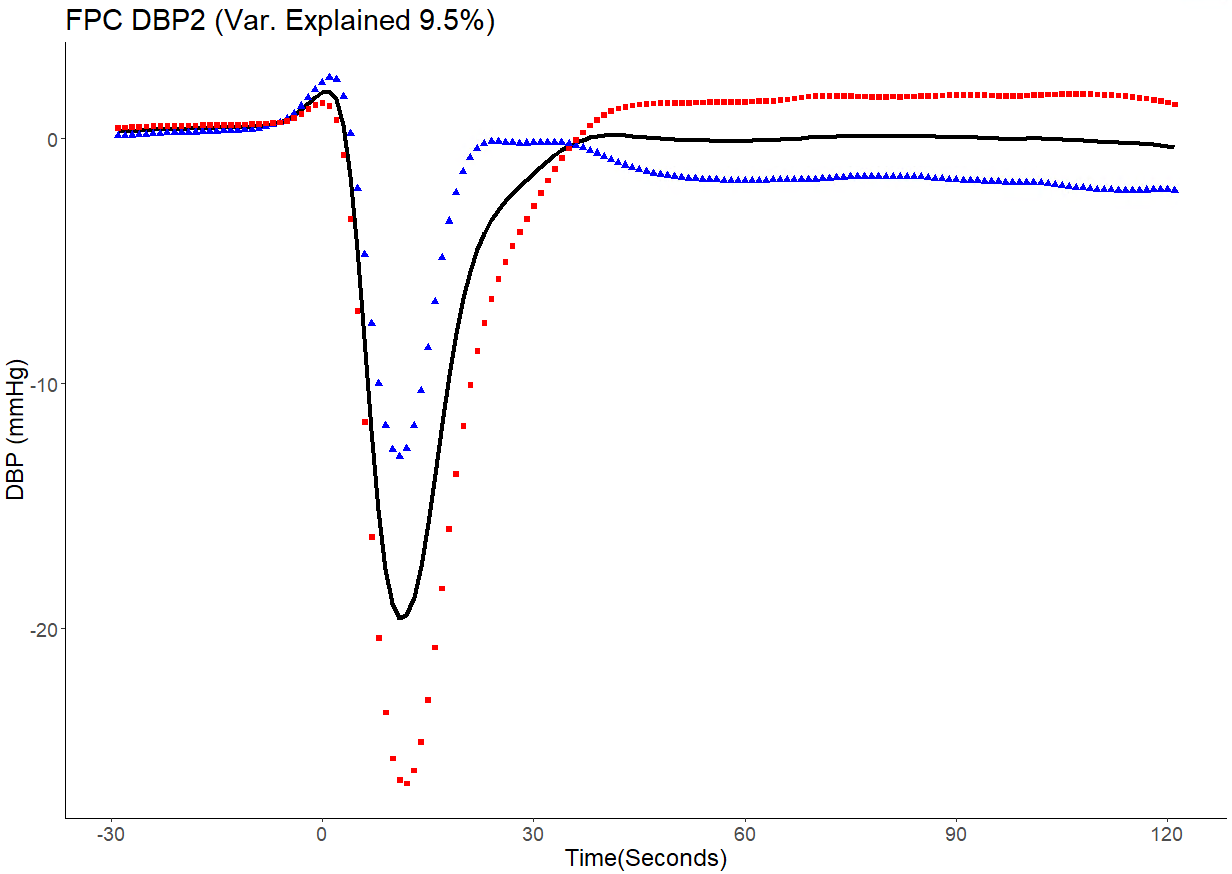


Supplementary Figure 5: How positive (red squares) and negative (blue triangles) FPC scores on the second principal component for DBP perturb the mean curve (black solid line) on a validation clinical cohort.

Figure Title: How FPC DBP2 impacts on the Mean DBP curve


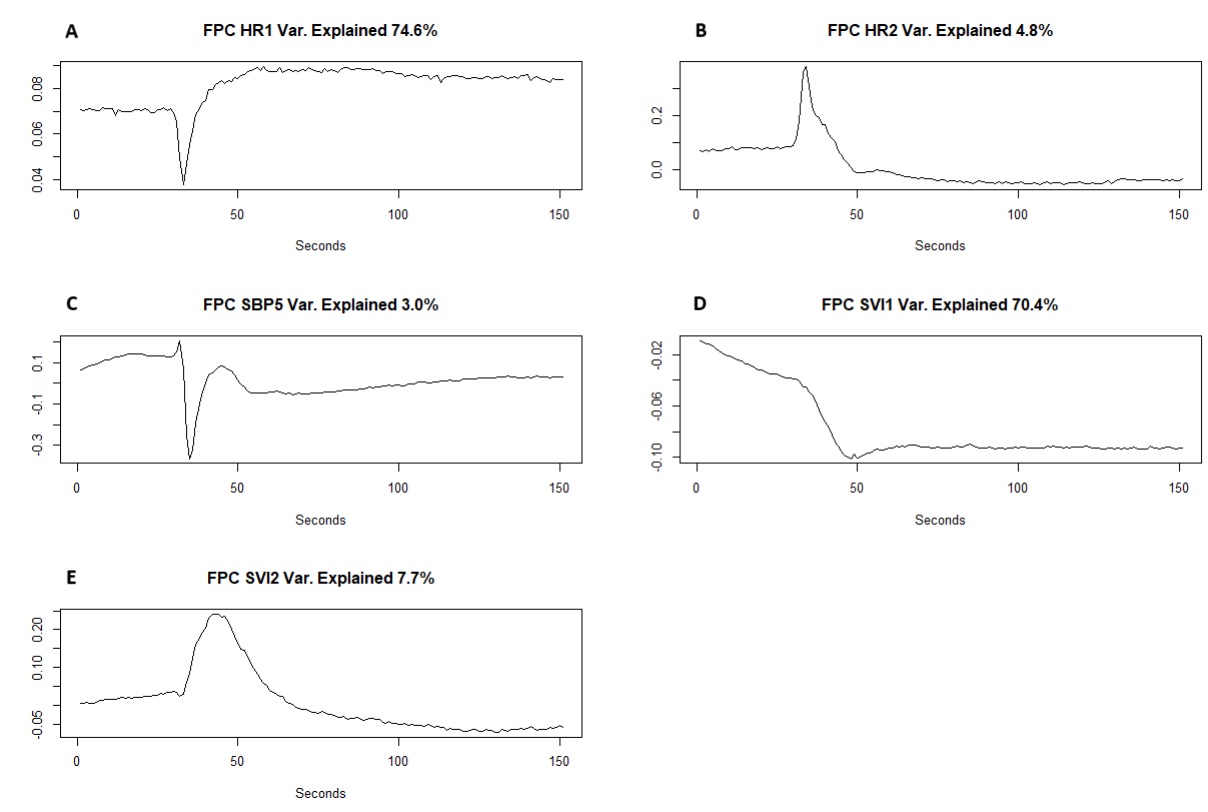


Supplementary Figure 6 Eigenfunctions associated with all-cause mortality, cause specific mortality and incident CVD. Figure 6 A Eigenfunction for FPC HR1 associated with all-cause mortality, other mortality and incident CVD.

Figure 6 B Eigenfunction for FPC HR2 associated with circulatory system mortality (positive association) and cancer mortality (negative association)

Figure 6 C Eigenfunction for FPC SBP5 associated with all-cause mortality and other mortality.

Figure 6 D Eigenfunction for FPC SVI1 associated with incident CVD and other mortality

Figure 6 E Eigenfunction for FPC SVI2 associated with cancer mortality.

Figure Title: Eigenfunctions associated with all-cause mortality, cause specific mortality and incident CVD
